# Translational Applications of Extracorporeal Shock Waves in Dental Medicine: A Literature Review

**DOI:** 10.3390/biomedicines10040902

**Published:** 2022-04-14

**Authors:** Abdulmonem Alshihri

**Affiliations:** Department of Prosthetic Dental Sciences, College of Dentistry, King Saud University, Riyadh 11545, Saudi Arabia; monem.alshihri@post.harvard.edu

**Keywords:** shock wave therapy, dentistry, oral, maxillofacial, sialolithiasis, TMD, bone defect, regenerative

## Abstract

Extracorporeal shock wave therapy (ESWT) has been studied and applied extensively in medical practice for various applications including musculoskeletal, dermal, vascular, and cardiac indications. These indications have emerged from primary ESWT use in treating urolithiasis and cholelithiasis. Likewise, dental medicine has had its share of utilizing ESWT in various investigations. This review aimed to provide an up-to-date summary of ESWT use in preclinical and clinical dental medicine. There is growing interest in ESWT use stemming from its non-invasiveness, low cost, and safe qualities in addition to its proven regenerative biostimulating aspects. Targeted tissue and parameters of ESWT delivery continue to be an integral part of successful ESWT treatment to attain the clinical value of the anticipated dose’s effect.

## 1. Introduction

An extracorporeal shock wave is a low-frequency, high-energy, pulsating sonic wave of short duration, generated outside the body. It was initially used around 1980 in urology [1]. From then on, a wide range of preclinical and clinical investigations with regenerative purposes have been reported. The indications of extracorporeal shock wave therapy (ESWT) evolved to treat bone and other musculoskeletal conditions such as tendinitis, nonunion fractures, aseptic necrosis of the bone, and myofascial disorders [2].

ESWT gained growing interest from researchers and clinicians in expanding the areas of potential use. This was fundamentally for its non-invasive, safe, low-cost, and fast application. A series of studies showed a positive effect of ESWT in different tissues such as cardiovascular [3], neural [4], and skin [5]. Based on the therapeutic indication and devices used, there are two types of shock waves, focused and radial, that can be favored depending on the indicated target depth and surface area. Radial waves reach a larger surface area and have less penetration depth compared to focused waves [6]. Devices that generate shock waves have different mechanisms of producing a waveform via electrohydraulic, piezoelectric and electromagnetic, and pneumatic stimulation. All methods can generate focused shock waves, while some electrohydraulic and electromagnetic devices can emit radial shock waves [6,7]. The shock wave involves a sharp increase in pressure that results in mechanical pressure and tension forces on the targeted tissue [7,8]. Radial shock therapy is considered a second-generation shock wave therapy. In addition, because of its lower energy and more linear pressure with less penetration depth—compared to focused—it arguably is safer [9,10]. The safety and efficacy of ESWT, regardless of its propagation pattern and waveform, is primarily based on a dosing recipe of fundamental factors including the total number of shocks, energy flux density measured in mJ/mm^2^, and the rate in Hz (shocks per second) [6,7]. Other variables, such as frequency of treatments (i.e., rounds of exposure) and interval of dose to effect, are vital to ESWT. The safety–efficacy and dose–effect parameters can metaphorically be simulated to a dose of a medication that is specifically tailored to treatment [11,12]. ESW travels through the tissue to the targeted site, where physical stimulation translates into biological outcomes. This response is an activation of cellular cascade events. Shock waves cause an increase in cell permeability and lead to the release of cellular proteins such as transforming growth factor-beta1, bone morphogenetic proteins, and other membrane-bound proteins [13,14]. It induces microscopic circulation and metabolism at the site [15,16,17]. It was reported that the ESW mechanotransduction has a molecular effect, triggering the release of cytokines, growth factors, and oxygen radicals [18]. These initial responses activate cellular biological reactions to proliferate, migrate, and differentiate, enhancing healing and promoting regenerative capability [12,16,19]. The positive regenerative effect of shock wave therapy is the leading cause for its recent interest and use [17]. It is possible to have an insignificant effect [20] or no effect using ESWT [21,22,23]. It is often related to ESWT parameters used in dose–effect settings. Therefore, it is vital that ESWT be applied to be valid, approved, and monitored [24,25]. Improper ESWT handling and lack of scientific support could induce injury or damaging effects with more destructive energy levels to different tissues of different impedances [26]. For instance, the lungs have low-density tissue mass and should be out of the wavefield for risk of tears or pneumothorax [26,27,28].

Other complications that vary in their severity have been reported. For instance, osteonecrosis of the humeral head [29], fascial ruptures [30,31] and acute myocardial infarction [32] were described during the course of ESWT.

ESWT is contraindicated in pregnant women [33] and in patients using anticoagulants (to avoid hemorrhage). ESWT application might pose a risk for bacteremia, and around tumors (tumor growth), where in either condition, ESWT has also shown an adjunctive positive effect. Therefore, appropriately trained professionals and internationally standardized care delivery should be followed [34]. The main side effects when using ESWT could include pain, skin erythema, and superficial hematoma [7].

Moreover, the noise associated with the use of ESWT could potentially be of harm to the hearing function of individuals treated as well as to the staff. In that aspect, a number of studies has investigated the effect of ESWT on the cochlear and auditory function of patients who had ESWT. Some clinical studies showed no hearing impairment was associated with ESW during lithotripsy [35,36] and plantar fasciitis therapy [37]. On the other hand, a study showed that ESWT had a collateral potential hazardous effect on hearing function [38], which is of further importance when considering treatment in the oral and maxillofacial area due to the proximity of exposure to the ears. Despite the controversy in the effect of ESWT on hearing function, it is vital for care providers and patients interacting with ESW to follow precautionary and protective measures. It was shown that the staff who operate ESWT with frequent exposure had experienced hearing loss [38,39].

In dental medicine, in the oral and maxillofacial areas, ESWT can present itself to include most indications it was invented and investigated to serve. The complex and diverse anatomical and histological structures of the oral and maxillofacial area include glandular, bone/musculoskeletal, soft tissue, and tooth-related conditions. The effect of ESWT is dose-dependent and site-specific. For that, other applications and doses used in different parts of the body cannot be applied for another. Thorough experimental and clinical investigations are essential to determine the appropriate shock wave parameters, dose–effect, and safety–efficacy guidelines needed for optimal therapeutic results [24,26].

Because of the reported ESWT stimulating, regenerative, anti-bacterial, and anti-inflammatory possibilities, ESWT’s indications have expanded to dental medicinal uses as well [40]. Numerous applications that ESWT can be investigated for include sialolithiasis, bone remodeling/fracture, periodontal therapy, soft tissue defects, and squamous cell carcinoma. In the present review, ESWT was explored for its up-to-date applications in dental medicine. The discussed studies are highlighted focusing mainly on the therapeutic and biological effects of ESWT on the cells/tissues of interest.

## 2. Literature Review

The topics discussed in this review include salivary glands, temporomandibular joint disorder, bone defect healing, orthodontics, periodontics, endodontics, tooth-desensitization, squamous cell carcinoma, and facial soft tissue (i.e., skin). A summary of findings for the clinical and preclinical studies is shown in Table 1 and Table 2, respectively.

### 2.1. Salivary Glands

Sialolithiasis is a benign situation characterized by the development of stones inside the ductal system of the major salivary glands: the parotid, submandibular, and sublingual glands [63]. It affects 1.2% of the population and is most common in people aged 30–60 years old [64]. Such calculi are reported in the submandibular gland in 80–90% of known cases [65]. Several diagnostic tools and conservative interventions are used, such as salivary gland massage, nonsteroidal anti-inflammatory drugs (NSAIDs), and sialagogues. However, sometimes conservative management fails to control the condition; hence, further treatment is needed [63]. In a retrospective study on patients with sialolithiasis, it was stated that “although the use of ESW lithotripsy does not produce outstanding results, it has very few adverse effects and therefore can be deemed as the first treatment to preserve the gland instead of total salivary gland removal”. Additionally, observed pain and self-esteem improved following ESW lithotripsy sessions. Moreover, they reported a decline in the percentage of patients with obstructive syndrome and noticed that their results were better in the parotid gland than the submandibular glands because the densities of parotid stones were less than those of submandibular ones [41]. Similarly, it was suggested that ESW lithotripsy was a choice for non-palpable lithiasis or those seen endoscopically [63].

### 2.2. Treatment of Temporomandibular Joint Disorders (TMDs)

Although several methods are available for the treatment of TMD patients, the proper selection of treatment methods is crucial for better treatment outcomes [66]. During a clinical study on patients with myogenic or mixed TMD, comparing the efficiencies of ultrashort wave (UW) and ESWT in treating TMD, it was found that the visual analog scale (VAS) scores were reduced in both groups following therapy, with the ESWT group experiencing a decrease greater than the UW group [42]. Likewise, the painless maximum mouth opening (MMO) in both groups improved following therapy. This could be attributed to the analgesic effect of ESWT, which hinders the excitation and propagation of pain signals through non-invasive stimulation of cellular membrane and nerve endings. The authors reported that the improvement was greater in the ESWT group than in the UW group, indicating that ESWT is superior to UW for TMD treatment. Usage of shock waves comes with minimal tears in non- or sparsely perfused tissues, inducing tissue reperfusion by releasing growth factors and stem cell mobilization, likely to result in blood flow to the cells [67].

A study on a rat model discussed the protective properties of ESWT on rats’ chondrocytes and temporomandibular joint osteoarthritis [46], revealing the down regulation effects of ESWT on pro-inflammatory cytokine expression of tumor necrosis factor α (TNFα), interleukin-1β (IL-1β), interleukin-6 (IL-6), apoptosis, and tissue degradation. This explained its effects on the behavior of chondrocytes. As a result, ESWT played a protective role in temporomandibular joint osteoarthritis cartilage and the subchondral bone structures in the monosodium iodoacetate (MIA)-stimulated chondrocytes and the MIA TMD model in rats.

Myofascial pain can be a major symptom associated with TMD. A study concluded that ESWT outperformed other therapies in aspects of pain intensity and pain threshold reduction in patients suffering from myofascial pain syndrome in the neck and shoulder following the intervention [68].

### 2.3. Fracture/Bone Defect Healing

The mandible is the most commonly injured and fractured bone in the maxillofacial area (52.9%). Such fractures are primarily managed with either open, closed reduction, or a combination of both techniques [69].

Fracture healing is a highly complex process affected by various biological and biomechanical factors. Post-operative risk of complications remains a significant drawback following oral and maxillofacial surgeries. Patients usually experience pain, trismus, edema, and infection, all of which negatively affect their life quality and psychological well-being [43]. According to the same study, ESWT is a safe and efficient treatment for fracture healing because it can enhance bone mass density, promote blood flow and metabolic activity in the adjacent soft tissue, and play a key role in pain relief [43]. In addition, an animal trial on rabbits was conducted to investigate changes in the quality of osseous regeneration and composition throughout the reparation of post-operative bone defect [47]. The authors noted that the bone defect regeneration happens due to the diversifying mineral deposits in the trabecular bone throughout all levels when using ESWT with a constant maximum wave front pressure. The newly formed bone tissue is structurally identical to the undamaged bone tissue, as the maximal pressure increases from 1.2 to 1.6 Bar [47]. Direct effect of ESWT on bone healing and acceleration of new bone formation could also reduce the possible collateral complications. In that context, a pilot study on rats indicated that combined intermaxillary fixation and ESWT reduces the duration of improvement in subcondylar mandibular fractures [70] ESWT could reduce the concomitant complications of lengthy fixation such as ankylosis, fibrosis, and hypomobility [70].

Furthermore, in a distraction osteogenesis (DO) rabbit model, a study proved that ESWT could stimulate growth factors and enhance the formation of new osteogenesis [48], but in an experimental study, it was observed that repeated application of low-energy flux density shock waves did not induce growth factors and angiogenesis, particularly in diabetic rats [49].

Additionally, an animal study on rabbits, which investigated the impacts of various dose levels of ESWT on repairing fractures using quantified stereological strategies, in addition to a radiological technique, reported that all application types of ESWT had a favorable impact on osteogenesis [50]. Comparably, in the DO study in a rat model, it was proved that following the distraction period, ESWT usage improves bone growth, development, and mineralization during DO. These effects are influenced by regulating the expression of bone-specific extracellular matrix proteins with no adverse reactions [51]. Furthermore, in a rat model study, repeated doses of ESWT on bony defects grafted with particulate allograft were investigated [52]. The results of radiographic and stereological analyses showed a higher bone density and more formation of new bone and connective tissue and capillaries at 8 weeks. It was concluded that the repeated ESWT induced the healing of grafted mandibular defects [52].

Osseointegration in dental implants is defined as an immediate, contextual, and functional relationship between structured viable bone and the surface of a functionally loaded implant. Therefore, dental implant osseointegration is considered as a natural system resembling fracture repair [71].

Dental implants have been commonly used in dental practice for the past few decades [72]. Currently, the implant accomplishes osseointegration when there is an accelerated interaction between both the implant and the bone it directly contacts [73].

The results of a hypothesis indicated that the biostimulator effect on resident human bone marrow-derived mesenchymal stem cells (HBMMSCs) could aid biological response to a more favorable outcome to achieve clinical success and limit side effects in implant integration [71]. The possible mechanism also includes the ESWT capacity to stimulate neoangiogenesis, mesenchymal stem cells (MSC) recruitment, cell proliferation and differentiation enhancement, and anti-inflammatory and antimicrobial effects [71].

In his letter to the Editor, Elisetti stated that ESWT, with its anti-inflammatory, osteogenesis, and antimicrobial advantages, could be a promising treatment option for peri-implantitis [74].

### 2.4. Orthodontics

In an animal study on a rat model, it was stated that novel methodologies to shorten prolonged procedures (which is considered the most significant drawback of orthodontic procedures) had been developed over the last few years [53]. ESWT is considered a non-invasive technology for this goal in addition to being a management protocol appropriate for clinical efficiency, particularly with the increased number of adult patients who seek orthodontic treatment and their interest in accelerating teeth movement to shorten the duration of treatment [53]. This study also demonstrated that “when compared to orthodontic force on its own, ESWT adds to the orthodontic power more than its double-speed of tooth movement in a rat model and created a considerable reduction in volumetric bone mineral density at the pressure side” [53].

A study on rabbits reported that ESWT was the origin of osteoclast internal migration in the maxillary area, with the biostimulative effect on alveolar bone marrow leading to fewer tartrate-resistant acid phosphatase (TRAP)-positive cells/area from 3 to 21 days [54]. The authors concluded that the usage of shock-wave therapy of focused 1000 impulses at energy flux density (EFD) 0.19 mJ/mm^2^, with a frequency of 5 pulses/second once weekly for a three-week duration, led to a significantly enhanced orthodontic tooth movement. Moreover, the use of ESW, with focused 500 or 1000 impulses, resulted in significantly enhanced bone formation, connective tissue, and blood capillaries formation, if applied once weekly for a three-week duration [54]. In addition, they concluded that implementing focused shock waves was more efficacious than unfocused shock waves in the growth of new bone and connective tissue for accelerating orthodontic tooth movement [54].

However, another study on orthodontic tooth movement in rats revealed that a single use of ESWT of focused 500 impulses at EFD 0.1 mJ/mm^2^, with a pulse rate of 5 pulses/second, applied when tooth motion was started, hindered tooth movement [55]. A single application of ESWT in the same study showed that, while there were beneficial consequences on osteoblastic activity in the bone renovation process, ESWT seemed to have no systemic effects on osteoclasts [55]. It was stated that at day 21, rats given ESWT had a higher rate of tooth movement, a lower volumetric bone mineral density, and a higher number of TRAP-positive cells/area on the pressure side [53]. An in vivo study on a rat model investigated the expression of cytokines (i.e., IL-1b, IL-6, RANKL, and TNF-a) in periodontal tissue of maxillary molars with a single application of ESWT (1000 pulse × 0.1 mJ/mm^2^) [56]. The study showed that the application of ESWT on orthodontically activated teeth induced an anti-inflammatory effect. Periodontal inflammatory cytokines were significantly reduced compared to the group that only received an orthodontic treatment without the ESWT [56].

However, a randomized clinical trial explored the effect of a single round of ESWT on orthodontic molar movement in the posterior–anterior direction, as well as tipping and rotation movement [22]. It revealed no difference between the treatment and control groups in terms of accelerating the tooth movement. The only significance the authors reported, as a minor clinical relevance, was in the buccolingual movement of the molars. Importantly, the study reported no complications or adverse events associated with ESWT during the study [22]. In the same controlled clinical trial, a different study reported the effect of ESWT on the stability of temporary anchorage devices (TADs) utilized for orthodontic movement. The research team concluded that the ESWT had no positive effect on improving the stability of TADs compared to placebo participants [44].

### 2.5. Periodontics

Periodontitis is an infectious disease caused by bacterial biofilm rather than planktonic pathogens [57]. A randomized clinical trial was conducted to investigate the effect of ESWT on periodontal parameters in patients’ periodontal profile measures of sulcus probing depth, gingival index, and plaque index [22]. The results showed no clinical difference in the outcomes of probing depth and gingival index using a single treatment of ESWT compared to the control group. The plaque index, however, showed significantly reduced measures in the ESWT group compared to the control group. This lower amount of plaque formation could be due to the anti-bacterial effect of ESWT on biofilm integrity. Nonetheless, there was no report of the qualitative effect of ESWT on the bacterial biofilm to exclude other confounding factors to this outcome. Participants in this study tolerated the ESWT with no complication or adverse reaction. Teeth and surrounding tissue in the field of the ESWT was monitored with no side effects or complications throughout the study period [22]. In another randomized controlled trial, the impact of a single treatment of ESWT on tooth mobility in the anterior mandibular area was investigated [45]. Tooth mobility is expected after orthodontic treatment due to the fact of ongoing bone remodeling, not a destructive periodontal disease. Periodontal probing depth and sulcular bleeding were also assessed. The findings of this clinical study showed a significantly faster reduction in tooth mobility [45]. The probing depth and bleeding on probing were significantly reduced in the ESWT group compared to the placebo group. This study’s stimulatory regenerative effect of ESWT was evident and could be concluded from the clinical findings reported. Neither complications nor adverse effects were reported in association with ESWT [45].

A literature review paper suggested that the use of ESWT as a non-invasive technique would be potentially implemented in periodontal treatment [75]. This was supported by its antimicrobial activity (particularly against periodontal pathogens), anti-inflammatory, analgesic, alveolar osteogenesis, and enhancing periodontal renovations and tissue regenerative characteristics with negligible or no documented adverse effects. A study on a humanized rat model discovered that the biofilm structure was disrupted when exposed to ESW, and its integrity was no longer preserved [57]. It was hypothesized that shock-wave exposure interrupted the extracellular polymeric substance (EPS), encompassing the biofilm, liberating bacteria, and potentially providing access to antimicrobial agents [57]. Furthermore, the authors showed that following the treatment, the animals treated with shock waves or antimicrobials alone did not recover from the disease significantly. On the other hand, the animals treated with a combined application of shock waves and antimicrobials recovered completely [57]. Periodontal ligament fibroblasts are key cells in periodontal tissue formation, regeneration, and remodeling. An in vitro study investigated the effect of different doses and energy flux densities of ESW on the expression of inflammatory mediators of human periodontal ligament fibroblasts [58]. The findings of this study showed no negative effect of ESW on cellular viability at 0.19 mJ/mm^2^ and 500 impulses. From a cytokines point of view, the ESW caused a higher expression in IL-6 and IL-8 and a decreased expression in TNF-α. Inflammatory cytokines can be expressed in pathological conditions, as in pro-inflammatory molecules in periodontal diseases as well as in cases of mechanical stimulation such as orthodontic movement. In relevance to the translational interpretation of these findings, ESWT could simulate a mechanical stimulation to periodontal ligament fibroblasts cellular activity and concomitant inflammatory reaction [58]. On the contrary, an in vivo study on a rat model investigated the expression of cytokines in periodontal tissue of maxillary molars with a single application of ESWT (1000 pulse, 0.1 mJ/mm^2^) [56]. The study showed that there were no differences in the periodontal profiles of cytokines (i.e., IL-1b, IL-6, and RANKL) in the ESWT group compared to the untreated group [56]. The authors indicated that the application of ESWT alone might not be sufficient in inducing the necessary threshold of periodontal cytokines. It was suggested that the ESWT “should always” be applied after the inflammatory process has been ignited by the intervention of concern, such as a surgical exposure, or orthodontic movement [56].

### 2.6. Endodontics

A randomized clinical trial investigated the effect of ESWT on the tooth pulp blood flow of mandibular incisors and canines in healthy patients [20]. After a single treatment of ESWT, pulpal blood flow was assessed (4 times/6 months) using a laser Doppler device. The findings of this study indicated a transient elevation of pulpal blood flow in the treated group with no significant difference compared to the control group over the study period of 6 months. No adverse events or complications were reported during the clinical trial [20]. Parameters of dose–response must be directed and tuned toward the translational goals in different pulpal pathological conditions such as pulpitis, pulpotomy, and regenerative endodontics.

Regarding cleaning and irrigation of root canals, an experimental study compared different irrigation methods and found that shock wave enhanced emission photoacoustic streaming (SWEEPS) demonstrated the greatest efficacy in cleaning the apical area in existence of a fractured instrument, with substantial differences from conventional irrigation [59]. While there is no substantial difference between passive ultrasonic irrigation and SWEEPS, the diagnostic and therapeutic significance of using a SWEEPS tip has the privilege of not requiring the laser tip to be taken to the apical area. Simply keeping the SWEEPS tip in the chamber is sufficient in doing its job; thus, it reduces preparatory missteps such as ledges or ultrasonic transportation [59].

### 2.7. Desensitization

Teeth desensitization, and, thus, pain reduction, is primarily based on isolating dental pulp from the external environment by modulating the dentinal tubule permeability. In an ex vivo study on extracted human molars, the dentinal tubules permeability was assessed by measuring the dentinal fluid flow (DFF) after applying a desensitizing agent, with or without ESWT [60]. It was found that the ESWT had a significantly additive effect of decreasing the DFF. It indicated the beneficial effect of ESWT in reducing the dentinal permeability by synergistically increasing the desensitizers tubular penetration. Yet, a clinical pain analysis study is needed to prove the principle and cause–effect relation.

Furthermore, using scanning electron microscopy (SEM), the desensitizer’s efficiency was compared with or without shock waves. The results confirmed that the dentinal penetration was doubled, and all tubules were occluded when adjunctive ESWT was used [60].

### 2.8. Squamous Cell Carcinoma

Squamous cell carcinoma is one of the most common malignant carcinomas of the head and neck [61]. In a rat study investigating the effect of different doses of ESWT on cells isolated from squamous cell carcinoma, it was reported that high energy levels of ESWT (>0.25 mJ/mm^2^) significantly suppressed cancerous cell proliferation and upregulated tumor cell apoptosis compared to a lower dose of ESWT (≤0.12 mJ/mm^2^). Further synergistic effects on suppressing the cancerous cells were found when high-energy (0.35 mJ/mm^2^) ESWT was combined with 5-FU anti-cancer drug molecules. It was explained that increased cell permeability and induced apoptosis facilitates anti-cancer drugs to tackle the tumor cells [61]. It was found that there was a more significant reduction in tumor volume and weight when using the combination of high-dose ESWT and 5-FU therapy than each one individually [61].

### 2.9. Facial Soft Tissue

Facial soft tissue must be critically approached in the process of repair after an injury or constructive interventions in cases such as cleft lip, facial trauma, burns, or tumor. Due to the fact of esthetic demands and limited skin grafting options, skin regeneration and scar reduction are of ultimate importance. In a large animal goat model, a study investigated the effectiveness of ESWT implementation in improving dermal thickness and vasculature and collagen production of facial skin [14]. They concluded that a single application of ESWT massively improved dermal thickness, the number and abundance of microvessels, and the amount of type 1 collagen in facial skin after four days of ESWT [14]. Such changes were not associated with the enhanced release of alpha smooth muscle actin (α-SMA) [14]. Moreover, an animal study on rabbits investigated the effect of ESWT on ear hypertrophic scar formation [62]. They concluded that both high and low ESWT could inhibit hypertrophic scar formation in hypertrophic scar tissues during the initial stages, enhancing scar elevation index and fibroblast density relative to the control group. They attributed this outcome to the effect of ESWT on inhibition of α-SMA expression of hypertrophic scar tissues [62].

## 3. Conclusions

In this narrative review, ESWT in dental medicine was discussed. Despite the limited number of homogenous studies available, ESWT has shown numerous advantages and potential benefits. There has not been a report of remarkable complications, substantial injury, or tissue damage in the studies discussed. It is evident that ESWT demonstrated regenerative, anti-inflammatory, antibacterial, and analgesic effects. These translational applications are certainly of great value to dental medicine patients and clinicians, especially with the increased demand, complexity, and cost of regenerative procedures. From the studies discussed in this review, it became clear that the type of ESW, dose, energy flux density, and number of cycles has yet to be optimized. It is crucial for researchers and clinicians to attempt to initiate and standardize the road map for homogenous predictable ESWT guidelines in oral and maxillofacial areas. Physical and dose–effect parameters are the cornerstone of establishing predictable positive biological and regenerative regimens. Therefore, more clinical studies are needed to determine the ideal amount of energy, dosage, and number of visits necessary for optimal therapeutic results. In addition, it is of further benefit to work with the manufacturing parties in designing the appropriate instrumentation and apparatus that suit the anatomical complexity of the oral and maxillofacial area.

## Figures and Tables

**Table 1:** Extracorporeal shock wave therapy (ESWT) applications in clinical studies in the oral and maxillofacial area.

	Reference	Year	Application Area	Therapeutic Goal	ESW Parameters	Outcomes	Study Type	Adverse Event
1	[20]	2015	Mandibular anterior teeth pulp	Pulpal blood flow	Focused, 1000 impulses, 5 Hz, 0.19–0.23 mJ/mm^2^	No identifiable effect	Comparative clinical trial	No side effect or complications were found
2	[21]	2016	Mandible	Bone marrow cell proliferation and growth factor expression	Focused, 1000 impulses, 4 Hz, 0.25 mJ/mm^2^, single session	No identifiable effect	Comparative clinical trial	Non-reported
3	[22]	2014	Mandibular molar area	Orthodontic tooth movement rate, periodontal profile measures	Focused, 1000 impulses, 5 Hz, 0.19–0.23 mJ/mm^2^	No significant effect	Comparative clinical trial	No side effect or complications were found
4	[41]	2018	Sialolithiasis in parotid and submandibular gland	Pain, obstructive syndrome, salivary gland infection, need for future surgery	Focused, 5000 impulses, 0.15–0.19 mJ/mm^2^, 2–4 sessions	Positive effect, decreased pain, obstruction, infection, and need for surgery	Retrospective clinical study, patient reported	Discomfort, dental/glandular pain, ecchymosis, cutaneous dermabrasion
5	[42]	2020	TMD/myofascial pain	TMJ/muscular pain, mandibular movement, joint noise, joint press, and disability index	Radial, 1000–1500 impulses, 8 Hz, 4 sessions	Positive effect, decreased pain, improved functional indexes of TMJ and mouth opening limit	Comparative clinical trial	Non-reported
6	[43]	2021	Mandible	Mandibular fracture	Focused, 4000 impulses, 0.35 mJ/mm^2^, 5 Hz, single session	Positive effect, pain reduction, increased bone density	Comparative clinical trial	Non-reported
7	[44]	2014	Mandibular molar area (orthodontic temporary anchorage devices (TADs))	Stability of orthodontic TADs	Focused, 1000 impulses, 5 Hz, 0.19–0.23 mJ/mm^2^	No identifiable effect	Comparative clinical trial	No side effect or complications were found
8	[45]	2015	Mandibular anterior teeth	Tooth mobility post-orthodontic treatment	Focused, 1000 impulses, 5 Hz, 0.19–0.23 mJ/mm^2^	Positive effect, reduced tooth mobility	Comparative clinical trial	No side effect or complications were found

**Table 2:** Extracorporeal shock wave therapy (ESWT) applications in preclinical studies related to the oral and maxillofacial area.

	Reference	Year	Application Area	Therapeutic Goal	ESW Parameters	Outcomes	Study Type
1	[14]	2020	Skin	Angiogenesis and collagen production	Focused, 1000 impulses, 4 Hz, 0.15 and 0.45 mJ/mm^2^, single session	Positive effect, induced dermal thickness, neovascularization, and collagen production	In vivo goat model
2	[23]	2019	Mandible	Distraction osteogenesis	Radial, 500 impulses, 5 Hz, 0.19 mJ/mm^2^, 3–6 sessions	No significant effect was found in the ESWs group vs. the control in bone density, formation, maturation, and vascularization	In vivo rabbit model
3	[46]	2019	Rat-derived chondrocytes, temporomandibular joint	Temporomandibular joint osteoarthritis	Focused, 500 impulses, 5 Hz, 0.068 mJ/mm^2^, 4 sessions	Positive effect, downregulation of pro-inflammatory cytokine expression, chondrocytes apoptosis, and TMJ tissue degradation	In vitro/in vivo rat model
4	[47]	2020	Mandible	Mandibular bone defect healing	Radial, 500 impulses, 1.2/1.6 pressure bar, 5 Hz, 0.19 mJ/mm^2^, 3 sessions	Dose-related positive effect on bone healing	In vivo rabbit model
5	[48]	2018	Mandible	Distraction osteogenesis	Focused, 500/1000 impulses, 5 Hz, 0.19 mJ/mm^2^, single session	Positive effect on induced bone mineral density and vascularization	In vivo rabbit model
6	[49]	2018	Mandible	Mandibular bone defect healing	Radial, 500 impulses, 5 Hz, 0.19 mJ/mm^2^, 3 sessions	No effect in non-diabetic, positive effect in diabetic animals for bone density, new bone formation, connective tissue, and neovascularization	In vivo rat model
7	[50]	2017	Mandible	Distraction osteogenesis	Focused, 500/1000 impulses, 4 Hz, 0.19 mJ/mm^2^, 2 sessions	Dose-related effect on consolidation of bone defect, significant induction of bone mineral density, new bone formation, and neovascularization	In vivo rabbit model
8	[51]	2019	Mandible	Distraction osteogenesis	Radial, 500 impulses, 0.18 mJ/mm^2^, 1 Hz, single session	Positive effect, induced bone formation, mineralization, mineral density, collagen deposition, and angiogenesis	In vivo rat model
9	[52]	2019	Mandible	Grafted (allograft) mandibular defects healing	Radial, 200 impulses, 5 Hz, 0.19 mJ/mm^2^, 3 sessions	Positive effect, accelerated graft healing, higher bone density, more new bone, connective tissue, and vessels formation	In vivo rat model
10	[53]	2022	Maxillary molars	Orthodontic tooth movement rate	Radial, 1000 impulses, 5 Hz, 0.1 mJ/mm^2^, single session	Positive effect, increased rate of tooth movement and bone remodeling	In vivo rat model
11	[54]	2021	Maxillary incisors	Orthodontic tooth movement rate	Radial/Focused, 500/1000 impulses, 5 Hz, 0.19 mJ/mm^2^, 3 sessions	Dose-related positive effect, increased rate of tooth movement and bone remodeling	In vivo rabbit model
12	[55]	2018	Maxillary molars	Orthodontic tooth movement rate	Focused, 500 impulses, 5 Hz, 0.1 mJ/mm^2^, single session	Increased bone cell activities, imbalanced-induced remodeling, hindered tooth movement rated	In vivo rat model
13	[56]	2015	Maxillary orthodontically activated molars	Periodontal cytokines levels	Radial, 1000 impulses, 5 Hz, 0.1 mJ/mm^2^, single session	Positive effect, anti-inflammatory effect, reduced periodontal cytokines expression	In vivo rat model
14	[56]	2015	Maxillary molars	Periodontal cytokines levels	Radial, 1000 impulses, 5 Hz, 0.1 mJ/mm^2^, single session	No identifiable effect	In vivo rat model
15	[57]	2019	Oral multispecies biofilm	Periodontitis	Customized shock wave model	Disrupted biofilm structure, significant reduction in multispecies biofilm when accompanied with antimicrobials	In vivo rat model
16	[58]	2016	Periodontal ligament fibroblasts	Periodontal cytokines levels	Radial, 100/300/500 impulses, 3 Hz, 0.05/0.10/0.19 mJ/mm^2^, single session	Dose-related effect on cytokine expression and cell viability	In vitro
17	[59]	2021	Extracted Molars, Root Canal	Efficiency of smear layer and debris removal	Shock wave enhanced emission photoacoustic streaming	Positive effect in removing the debris and smear layer	In vitro
18	[60]	2020	Extracted molars, root canal	Dentinal tubules seal, desensitizers penetration	Focused, micro-shock wave generator, piezo-electric actuator (100 m stroke, 10,000 G)	Positive effect, reduced dentin permeability, and enhanced dentinal tubule occlusion	In vitro
19	[61]	2021	Tongue squamous cells	Squamous cell carcinoma proliferation and tumor growth	Focused, 140 impulses. Cells: 0.1, 0.12, 0.14, 0.25, 0.35 mJ/mm^2^. Animals: 500 impulses, 0.05, 0.1, 0.3, 0.5 mJ/mm^2^, 4 sessions	Dose-related effect on cell viability, positive effect on suppressing cell proliferation, and inducing tumor cell apoptosis	In vivo mouse model
20	[62]	2018	Skin	Hypertrophic scar formation	Radial, 500 impulses, 8 Hz, 0.1, 0.2 mJ/mm^2^, 4 sessions	Positive effect in suppressing the hypertrophic scar formation, reduced scar elevation index, fibroblast density, and a-SMA	In vivo rabbit model
